# Influence of Material Type and Specimen Geometry on Polymerization Shrinkage of Resin-Based Cements: A 3D-DIC Multifactorial Study

**DOI:** 10.3390/ma19214538

**Published:** 2026-10-28

**Authors:** Aleksandra Mitrovic, Snežana Kirin, Svetlana Pelemiš, Nenad Mitrovic

**Affiliations:** 1 University of Criminal Investigation and Police Studies, Faculty of Forensic Sciences and Engineering, 11080 Belgrade, Serbia; aleksandra.mitrovic@kpu.edu.rs; 2 Innovation Center of Faculty of Mechanical Engineering, 11120 Belgrade, Serbia; skirin@mas.bg.ac.rs; 3 Faculty of Technology Zvornik, University of East Sarajevo, 75400 Zvornik, Bosnia and Herzegovina; svetlana.pelemis@tfzv.ues.rs.ba; 4 Faculty of Mechanical Engineering, University of Belgrade, 11120 Belgrade, Serbia

**Keywords:** polymerization shrinkage, resin-based cements, 3D digital image correlation, three-way ANOVA, dental materials

## Abstract

**Background/Objectives:** Polymerization shrinkage of resin-based cements critically affects the integrity and longevity of indirect dental restorations, yet the combined influence of material composition, specimen geometry, and measurement strategy remains insufficiently quantified. The aim of this study was to evaluate the individual and interaction effects of these parameters on shrinkage strain using a multifactorial approach. **Methods:** Shrinkage strain was evaluated using three-dimensional digital image correlation (3D-DIC) coupled with three-way ANOVA. Four commercially available dual-cured self-adhesive resin-based cements were analyzed in a 4 × 2 × 3 multifactorial design, with specimen thicknesses of 1 and 2 mm and specimen diameters of 3, 4 and 5 mm (*n* = 3 per group, 72 specimens). Strain was assessed in both circular sections at the material–mold interface and orthogonal linear sections. **Results:** Material type (*p* < 0.001) and its interaction with specimen thickness (*p* = 0.002) were identified as dominant factors governing strain, whereas specimen diameter showed no statistically significant effect (*p* = 0.460). Increasing thickness from 1 to 2 mm resulted in a rise in strain values (from 2.273% to 3.001%), with the highest strain observed for one of the cements at 2 mm thickness (4.107%). The circular section exhibited the highest sensitivity to thickness-dependent variations, while linear sections showed consistent but less pronounced trends. Post hoc analysis separated the cements into high-strain and low-strain subsets, and the grouping depended on the measurement section. **Conclusions:** Polymerization shrinkage is primarily governed by material properties and their interaction with layer thickness, rather than specimen size within the investigated range. A specimen thickness of 2 mm and circular section analysis are recommended for improved sensitivity and comparability in experimental studies. Clinically, minimizing cement layer thickness and selecting appropriate materials are essential for reducing shrinkage-induced stresses and improving the durability of dental restorations.

## 1. Introduction

In recent times, dual-cured resin-based cements (RBCs) have emerged for dental applications due to their ability to self-cure under indirect aesthetic polymeric or ceramic restorations [1,2]. When compared with traditional luting agents, modern RBCs exhibit superior marginal sealing, retentive capability, and adequate physical and mechanical properties. These include the enhanced fracture resistance of the restorations placed on top, resulting in an optimal aesthetic outcome [3].

The appropriate selection of RBCs is critical to the durability of dental restorative materials. Each type of ceramic system has a unique composition, necessitating suitable resin-based cements and cementation protocols. Dual-cured self-adhesive RBCs [4] were developed to streamline the resin bonding process, minimizing the steps and time required during dental bonding procedures. These cements offer the benefits of dual curing and ease of use, eliminating the need for the pretreatment of dental tissues. They are based on innovative technology that involves specific monomers, fillers, and initiators. These monomers are also responsible for the shrinkage stress and shrinkage strain observed within RBCs during the polymerization process [5,6]. Specifically, the strain observed during the transformation from monomer to polymer causes micro-leakage and significantly impacts the longevity of restorations due to cement degradation [7,8]. However, even with the advancement of materials, polymerization shrinkage remains a challenge [9].

Shrinkage strain depends on the thickness of the cement layer and is a significant source of shrinkage stress [10,11], which can cause micro-leakage and adversely affect the longevity of restorations due to cement degradation [12]. Furthermore, the thickness of the cement layer has been identified as a critical factor in the cementation process [13].

Various contact and non-contact techniques have been utilized to experimentally examine the polymerization shrinkage of dental composites. Current methodologies utilize the state-of-the-art 3D digital image correlation (3D-DIC) method as an optical sensor for detecting shrinkage strain [14,15,16,17,18,19,20]. Researchers use statistical methods to analyze and interpret obtained data related to the characteristics and performance of dental materials. For example, Pratap et al. [21] discuss the development of dental materials with improved mechanical properties, including reduced polymerization shrinkage and enhanced depth of cure. They emphasize the importance of statistical analysis in assessing these properties. Similarly, Zhang et al. [22] review the rheological and mechanical properties of resin-based dental materials, highlighting the need for statistical analysis to understand their behavior. Other studies [23] investigate the influence of different factors, such as filler type and size, on the mechanical properties of dental materials using statistical methods. However, further analysis is needed for a deeper understanding of dental materials, especially for a comparison of the different materials with multiple variables that influence mechanical properties.

Multifactorial analysis allows the main and interaction effects of several independent variables on a single outcome to be evaluated simultaneously. This type of multifactorial design has been successfully applied in dental research to disentangle the simultaneous influence of multiple clinically relevant variables on a single outcome [24].

Previous 3D-DIC studies on dental resin-based cements, including our own [14,15,25], quantified the shrinkage strain of individual materials or compared materials using a single, fixed specimen geometry, while DIC has more broadly become an established tool for full-field displacement and strain measurement in dental materials research [20]. To the best of our knowledge, the combined and interactive effects of material type, specimen thickness and specimen diameter on DIC-measured shrinkage strain, as well as the dependence of the result on the location of the evaluation line (circular section at the material–mold interface versus linear sections across the specimen), have not yet been systematically examined. This information is needed both for interpreting the role of cement layer thickness and for standardizing DIC test protocols, so that results obtained in different laboratories can be compared. The novelty of the present study therefore lies in combining 3D-DIC with a multifactorial design and three-way ANOVA to separate the main and interaction effects of these factors.

The aim of this paper is to apply a multifactorial analytical approach to compare the mechanical properties of dental materials, that is, to establish whether there are statistically significant differences between factors, material type, sample thickness, and sample diameters with respective strain values measured using the 3D digital image correlation method. In this study, the 3D-DIC method was employed as an optical technique to detect shrinkage strain in composite cements immediately after photo-polymerization.

## 2. Materials and Methods

In this paper, four commercially available self-adhesive resin-based dual-curing composite cements were observed: Maxcem Elite (Kerr, Brea, CA, USA), RelyX U200 (3M ESPE, Seefeld, Germany), Multilink Automix (Ivoclar Vivadent, Schaan, Liechtenstein) and SeT PP (SDI, Bayswater, Australia). Cylindrical molds with diameters of 3 mm, 4 mm and 5 mm, combined with two different values of thickness, 1 mm and 2 mm, were prepared for each composite. For each group, three samples were prepared. In total, 72 samples were prepared by filling ring-type molds. Since the specimens were not bonded to any substrate, no primer or adhesive pretreatment was applied. Each experimental group corresponded to one combination of material type (4 levels), specimen thickness (2 levels) and specimen diameter (3 levels), giving a 4 × 2 × 3 multifactorial design with three specimens per combination, as summarized in Table 1 [26,27].

The ring-type molds were made of polytetrafluoroethylene (PTFE, Teflon, Zeus Industrial Products, Orangeburg, SC, USA), whose low surface energy and non-adhesive character minimize adhesion and friction between the cement and the mold wall; no separating agent or other surface treatment was applied. The dimensions of each mold were verified with a digital caliper with an accuracy of 0.01 mm. Each mold was placed on a polyester (Mylar, Mylar Specialty Films US LP, Hopewell, VA, USA) strip on a glass slide and filled with the cement in a single increment, using the automix tip and a metal hand instrument, while avoiding entrapment of air bubbles. A second polyester strip was then placed over the top surface and gently pressed to distribute the material evenly, and was removed after a few seconds. The specimens remained in the molds throughout light curing and image acquisition, so that the lateral surface of each specimen was confined by the mold wall while the top surface was free. All specimens were prepared and tested at room temperature, following an identical sequence and timing of preparation, irradiation and image acquisition for every specimen to ensure repeatability.

Light curing was performed with an LED curing unit (LEDition, Ivoclar Vivadent, Schaan, Liechtenstein) with an irradiance of 500–900 mW/cm^2^ and an emission spectrum in the range of 430–490 nm. The exposure time was 20 s, as recommended by the manufacturers of all four cements, applied as a single continuous exposure. The light guide was fixed in a holder beneath the mold, so that the bottom surface of the specimen was irradiated through the glass slide and polyester strip at a constant distance of 1 mm for all specimens, while the opposite (top) surface faced the DIC cameras.

The von Mises strain field was measured using the 3D optical system Aramis 2M (GOM, Braunschweig, Germany) based on the 3D-DIC method [28]. The system consisted of two digital cameras with a resolution of 1600 × 1200 pixels equipped with 50 mm lenses, positioned at a distance of 28 cm from the specimen, with a measuring volume of 25 mm × 20 mm × 10 mm. Prior to the measurements, the system was calibrated with the manufacturer’s calibration object, and the calibration deviation was within the range prescribed by the manufacturer (0.01–0.04 pixels). A stochastic pattern was applied to the top surface of each specimen by spraying first a thin layer of matte white paint and then a fine black speckle pattern; the specimens were covered with a dark box while the paint dried, to eliminate the influence of ambient light. 3D-DIC image acquisition was performed immediately before and after light-curing polymerization, as previously described [14,15,25]. The reference image (Stage 0) was recorded immediately before light curing, and the deformed image (Stage 1) immediately after the end of the 20 s light exposure. The reported values therefore represent the immediate shrinkage strain developed during the light-activated phase of polymerization, relative to the uncured state; the further (self-cure) shrinkage occurring after this point was not included in the analysis. Displacement and strain fields were computed in the Aramis v6.2 software, and the von Mises (equivalent) strain was calculated from the components of the strain tensor. For each specimen, the mean von Mises strain along each section was used as one observation in the statistical analysis. Experimentally measured von Mises strain values were used to analyze the influence of material type, sample thickness, and sample diameter. For every sample, the von Mises strain field was measured. Analysis of the von Mises strain fields was performed using three sections (Sections 0, 1 and 2). Section 0 is a circular evaluation line that follows the periphery of the specimen surface at the material–mold interface. Sections 1 and 2 are two straight evaluation lines that pass through the center of the specimen surface and are perpendicular to each other, crossing at the specimen center. The diameter of Section 0 and the length of Sections 1 and 2 correspond to the diameter of the sample. The positions of these sections on the measured strain field are illustrated in our previous study that used the same procedure [14].

The following null hypotheses were established:

**H0_1_.**  *The type of composite cement material will not have a significant effect on the sample strain value.*

**H0_2_.**  *The sample thickness will not have a significant effect on the sample strain value.*

**H0_3_.**  *The sample diameter will have no significant effect on the sample strain value.*

**H0_4_.**  *The interaction of material type and sample thickness will not have a significant effect on the sample strain value.*

**H0_5_.**  *The material type and the sample diameter interaction will have no significant effect on the sample strain value.*

**H0_6_.**  *Material type, sample thickness, and sample diameter interaction will have no significant effect on the sample strain value.*

**H1.**  *The way the data are collected from the sample affects the conclusion of the material strain.*

The experimentally obtained data were processed using the IBM SPSS Statistics 26 software package (IBM, Armonk, NY, USA). As four different materials, two different sample thicknesses, and three sample diameters were applied in the experiment, analysis of variance (ANOVA) was applied for statistical analysis. ANOVA is often used for the analysis of material test data, and in this study it was used to establish whether there are statistically significant differences between material type, sample thickness, sample diameters, and strain values. The ANOVA method uses the F statistic to test the null hypothesis, which claims that there is no difference in characteristics between the observed groups. The results of ANOVA analyses with *p*-values smaller than 0.05 (*p* < 0.05) were considered statistically significant. The ANOVA F test assumes homogeneity of variance of the observed groups because the failure of this assumption alters Type I error rates, that is, rejection of the true null hypothesis [29]. When variances are homogeneous, the F test is the optimal test [30]. A three-way ANOVA was performed to investigate the effect and contribution of each material type, sample thickness and diameter, as well as the mutual influence of independent variables on strain values.

## 3. Results

The dependent variable is the von Mises strain in Sections 0, 1 and 2, and statistical analysis is presented for each section. Independent variables are material type, sample thickness, and sample diameter and consist of four, two, and three categorical independent groups, respectively, as shown in Table 1.

Strain data on independent variables were obtained experimentally. The mean and standard deviation of the strain data on Section 0 for the four considered materials, two categories of sample thickness, and three different diameters are shown in Appendix A. Conditions for the application of the three-way ANOVA in Section 0 were met, i.e., there was no relationship between the observations in each group or between the groups themselves, so the independence of observations was satisfied. The sample was without significant outliers, and homogeneity of variances for each combination of the groups of the three independent variables were satisfied. Table 2 presents Levene’s test, which tests the null hypothesis that the error variance of the dependent variable is equal across groups.

A Kolmogorov–Smirnov test indicates that the strain values on Section 0 follow a normal distribution, D(72) = 0.102, *p* = 0.06.

The primary goal of running a three-way ANOVA is to determine whether there is a three-way interaction between three independent variables. Essentially, a three-way interaction tests whether simple two-way material type–sample thickness interactions differ between the diameter range [31]. The importance of the influence of material type, sample thickness and diameter on the strain on Section 0 can be seen through the *p*-value of the tests of between-subjects effects (Table 3). The obtained values show a statistically significant influence of material type (*p* < 0.001) and sample thickness (*p* < 0.001), as well as their combination (*p* = 0.002). Therefore, the hypotheses H0_1,_ H0_2_, and H0_4_ were rejected, that is, the alternative claims are valid:

- The material type will have a significant effect on the sample strain values.
- The sample thickness will have a significant effect on the sample strain values.
- The interaction of material type and sample thickness will have a significant effect on the sample strain value.

The diameter of the sample did not significantly affect the measured strain value (*p* = 0.460 > 0.05). The combination of material type and sample diameter did not have a statistically significant effect on the measured strain (*p* = 0.058 > 0.05), nor did the combination of material type, sample thickness and sample diameter (*p* = 0.492 > 0.05). Therefore, hypotheses H0_3_, H0_5_, and H0_6_ were confirmed. The combination of all three variables, material type, sample thickness and sample diameter, observed together, does not significantly affect the measured strain (*p* = 0.476 > 0.05). The obtained results show the influence of only two independent variables on the dependent variable, i.e., the strain value.

The mean strain values on Section 0 for different materials are given in Table 4. The highest mean strain was obtained for the RelyX U200 material and the lowest for the Maxcem material.

The sample thickness has a statistically significant effect on the strain value. A higher strain value was obtained in samples with a thickness of 2 mm. The mean strain values on Section 0 for different thicknesses are given in Table 5.

The mean strain values on Section 0 for different diameters are given in Table 6. It was mentioned before that these differences are not statistically significant.

**Figure 1:**
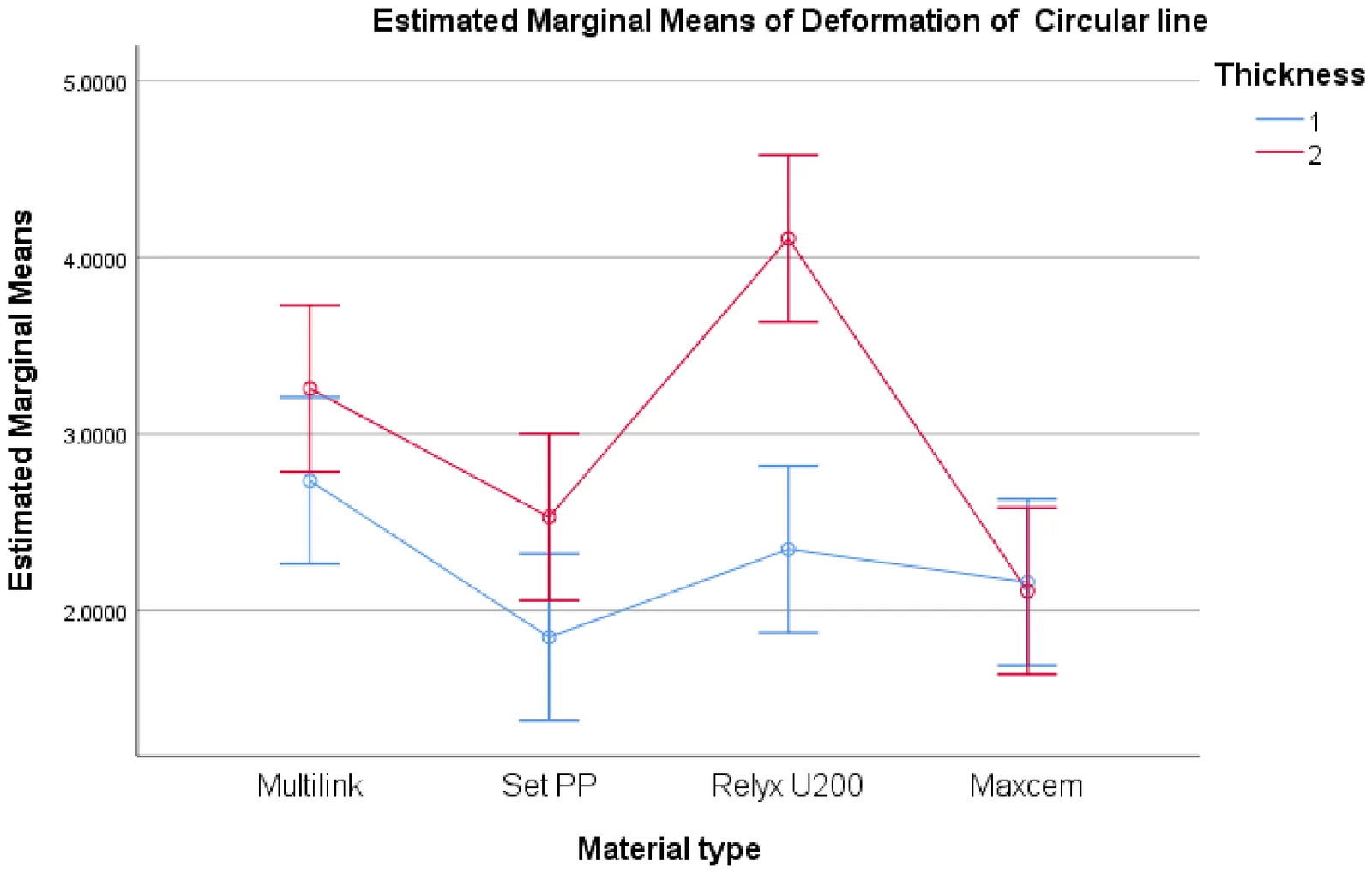
Estimated marginal means of strain on Section 0 for sample thicknesses of 1 and 2 mm with error bars for 95% confidence interval.

The combination of material type and sample thickness was found to have a statistically significant effect on measured strain. The mean strain values depending on the combination of material type and sample thickness are shown in Table 7 and Figure 1. These differences are statistically significant.

The highest strain value was obtained for RelyX U200 materials with 2 mm sample thickness (4.107%). The next highest strain value was obtained for the Multilink material, with a sample thickness of 2 mm (3.257%). The lowest strain value was obtained for the SeT PP material, with a sample thickness of 1 mm (1.849%).

**Figure 2:**
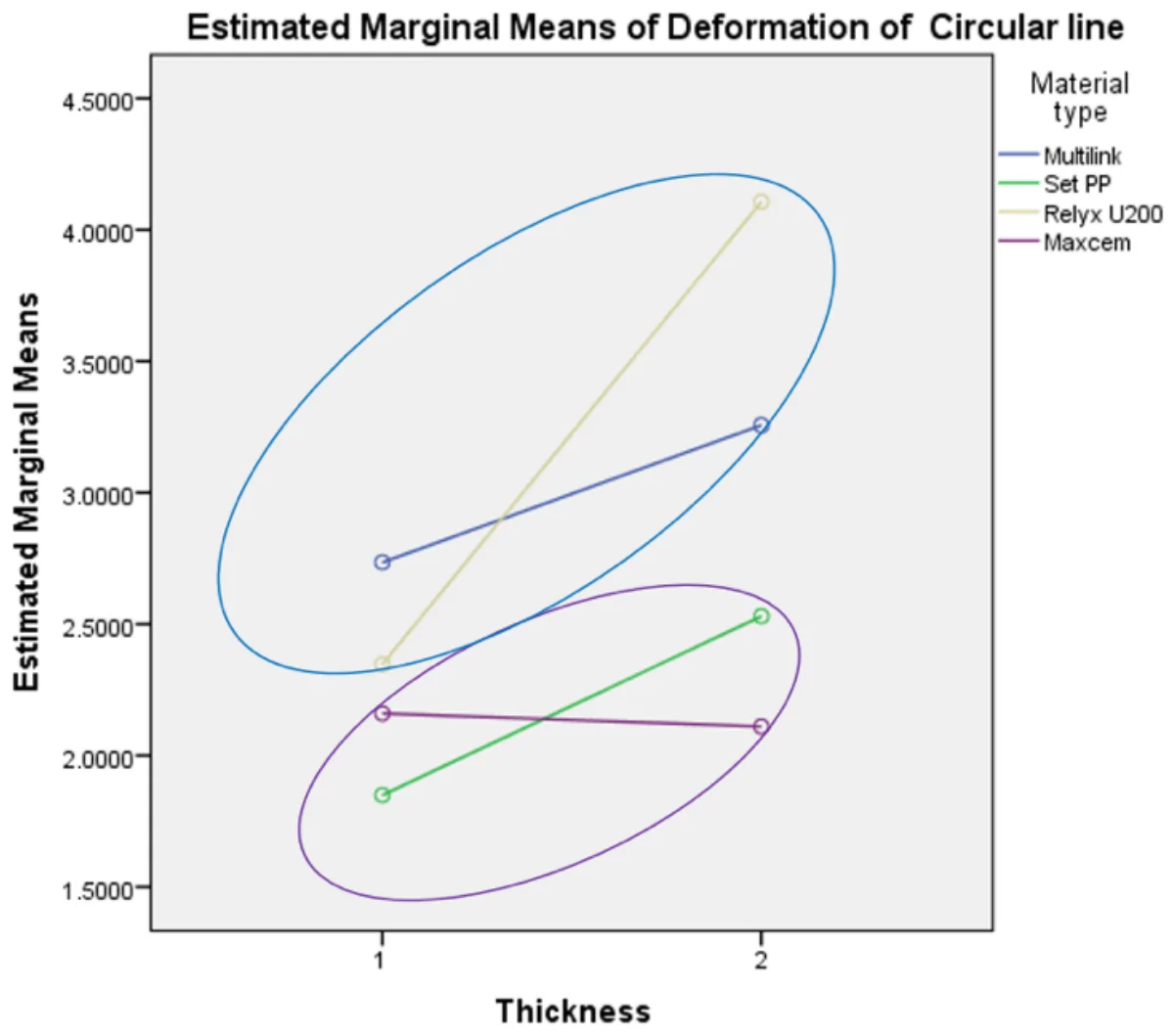
Homogeneous subsets of estimated marginal means of strain values on Section 0.

Estimated marginal means of the strain on Section 0 are given in Figure 1 and Figure 2.

The three-way ANOVA test does not show how the means differ, so for multiple comparisons of means, Tukey’s HSD test was applied, which compares all possible pairs of means. This analysis is very useful when there is a categorical factor and a continuous response, assuming that each set of data has equal variance (Tukey’s comparison procedure). This helps to determine whether the means of the two groups of properties are different. Multiple comparisons of strain values on Section 0 in relation to material type and sample thickness are given in Table 8.

Using Tukey’s HSD post hoc test, two groups of homogeneous subsets were obtained depending on the material type and sample thickness, as shown in Table 9 and Figure 2. Two homogeneous subsets with respect to the strain on Section 0 were obtained:

1. Maxcem and SeT PP, where the strain difference is not statistically significant.
2. Multilink and RelyX U200, which do not differ statistically significantly.

**Figure 3:**
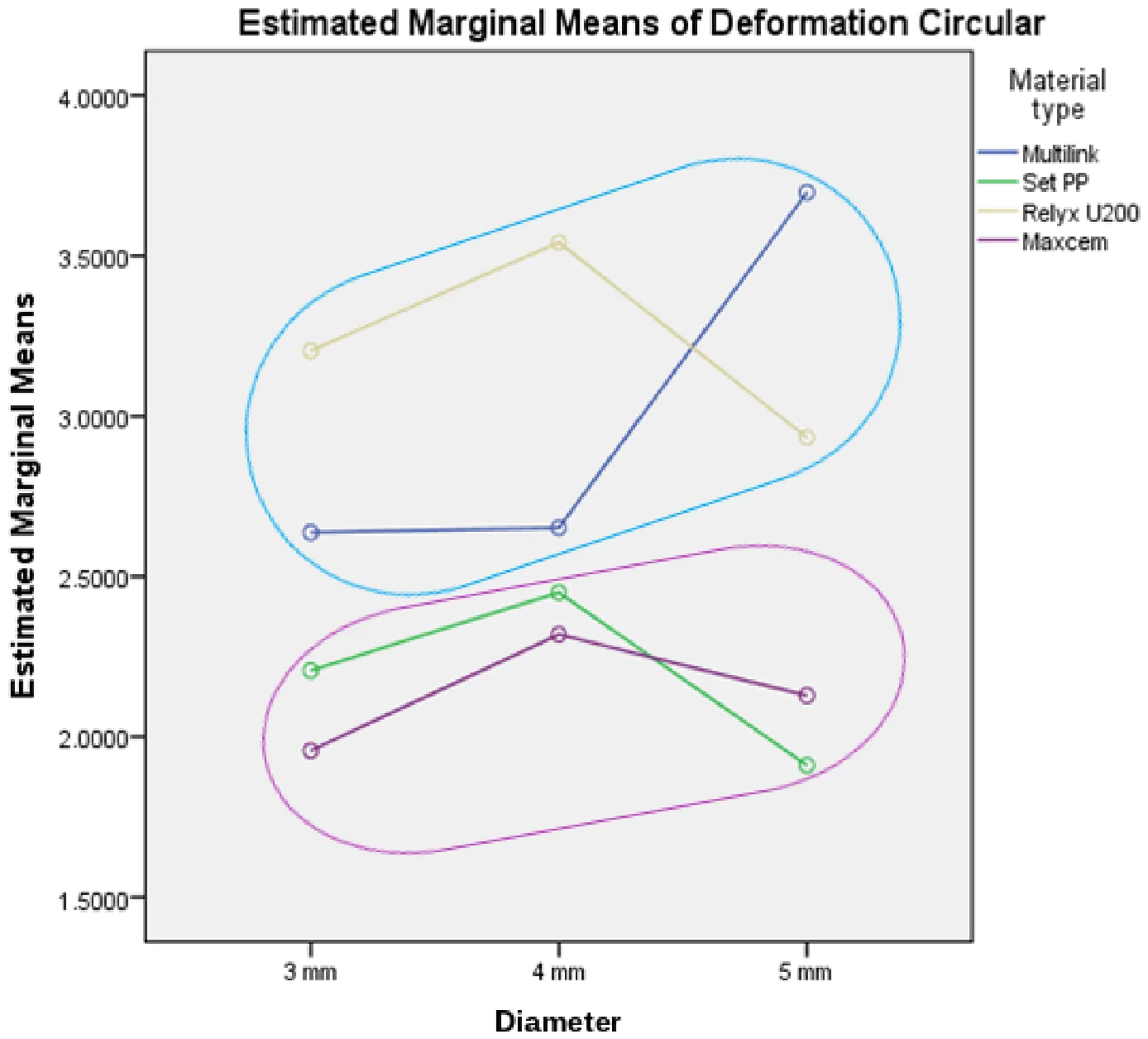
Estimated marginal means of strain values on Section 0 related to sample diameter.

Similarities and differences related to the strain values on Section 0 should be observed in the context of defining the material type and the sample thickness, as the RelyX U200 and Multilink materials have similar strain values, as well as the SeT PP and Maxcem materials. It was shown earlier in the paper that the sample diameter does not have a statistically significant effect on the sample strain values for the four materials observed. Figure 3 shows the strain values obtained for different sample diameters.

In order to determine the strain of the observed materials, in addition to the values on Section 0, the points on Sections 1 and 2 were also observed. The mean values and standard deviation of the data on Sections 1 and 2 for the four considered materials, two categories of sample thickness, and three different diameters are shown in Appendix B and Appendix C, respectively.

The strain on Section 1 is statistically significantly influenced by material type, as well as the combination of material type and sample thickness, while the individual influences of sample thickness and sample diameter are not statistically significant (Table 10). The obtained results show that the strain is statistically significantly dependent on the material type and sample thickness, regardless of whether a circular or straight line is observed.

**Figure 4:**
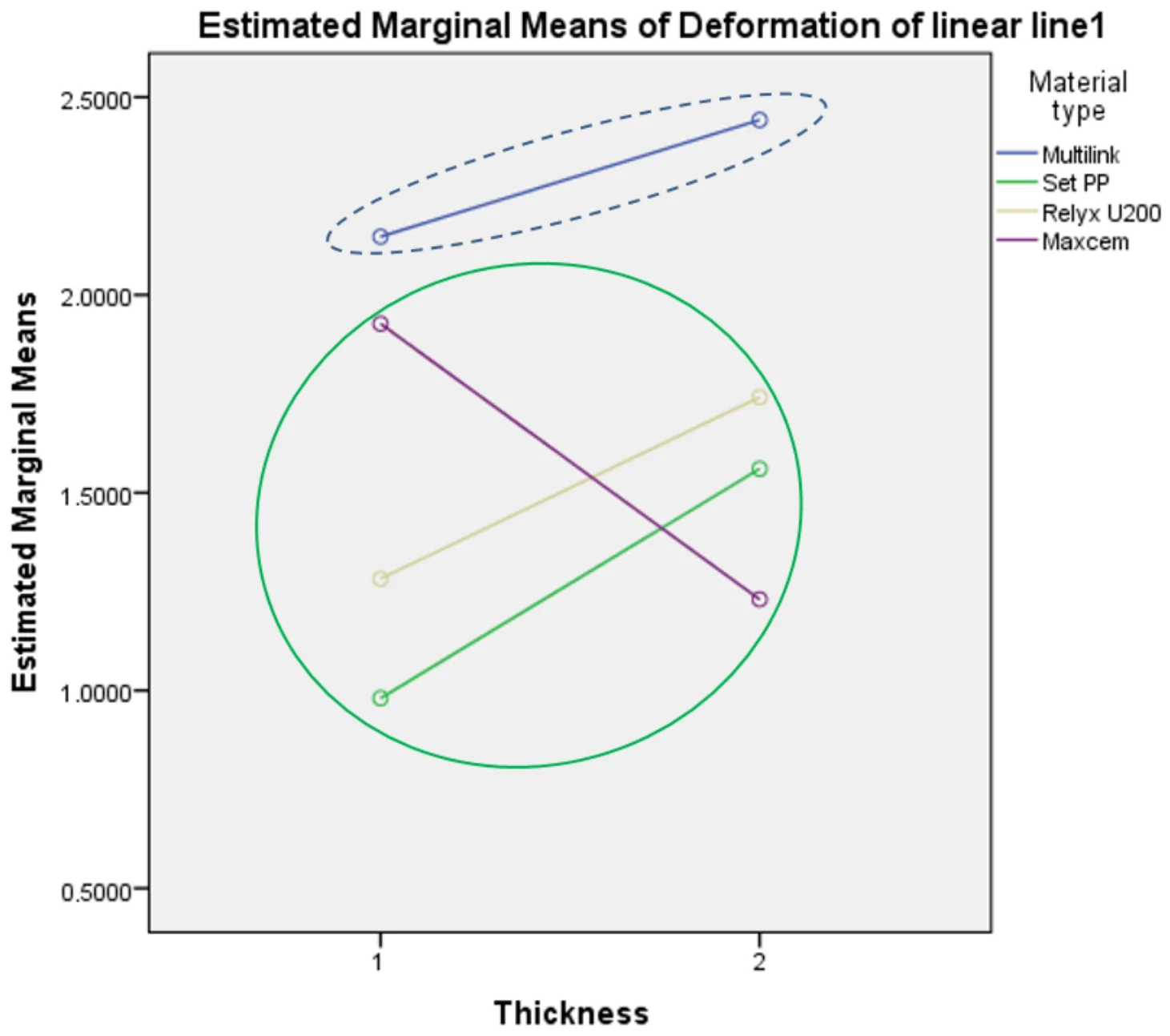
Homogeneous subsets of estimated marginal means of strain values on Section 1.

Using Tukey’s HSD post hoc test, two groups of homogeneous subsets were obtained depending on the material type and sample thickness, as shown in Table 11 and Figure 4. Two homogeneous subsets were obtained with respect to the strain on Section 1:

1. Maxcem, RelyX U200 and SeT PP, for which the difference in strain is not statistically significant.
2. Multilink, which does not differ statistically significantly.

Similar results were obtained by analyzing the strain on Section 2, i.e., the strain is statistically significantly affected by the material type, as well as the combination of material type and sample thickness, while the individual influences of sample thickness and sample diameter are not statistically significant, Table 12.

**Figure 5:**
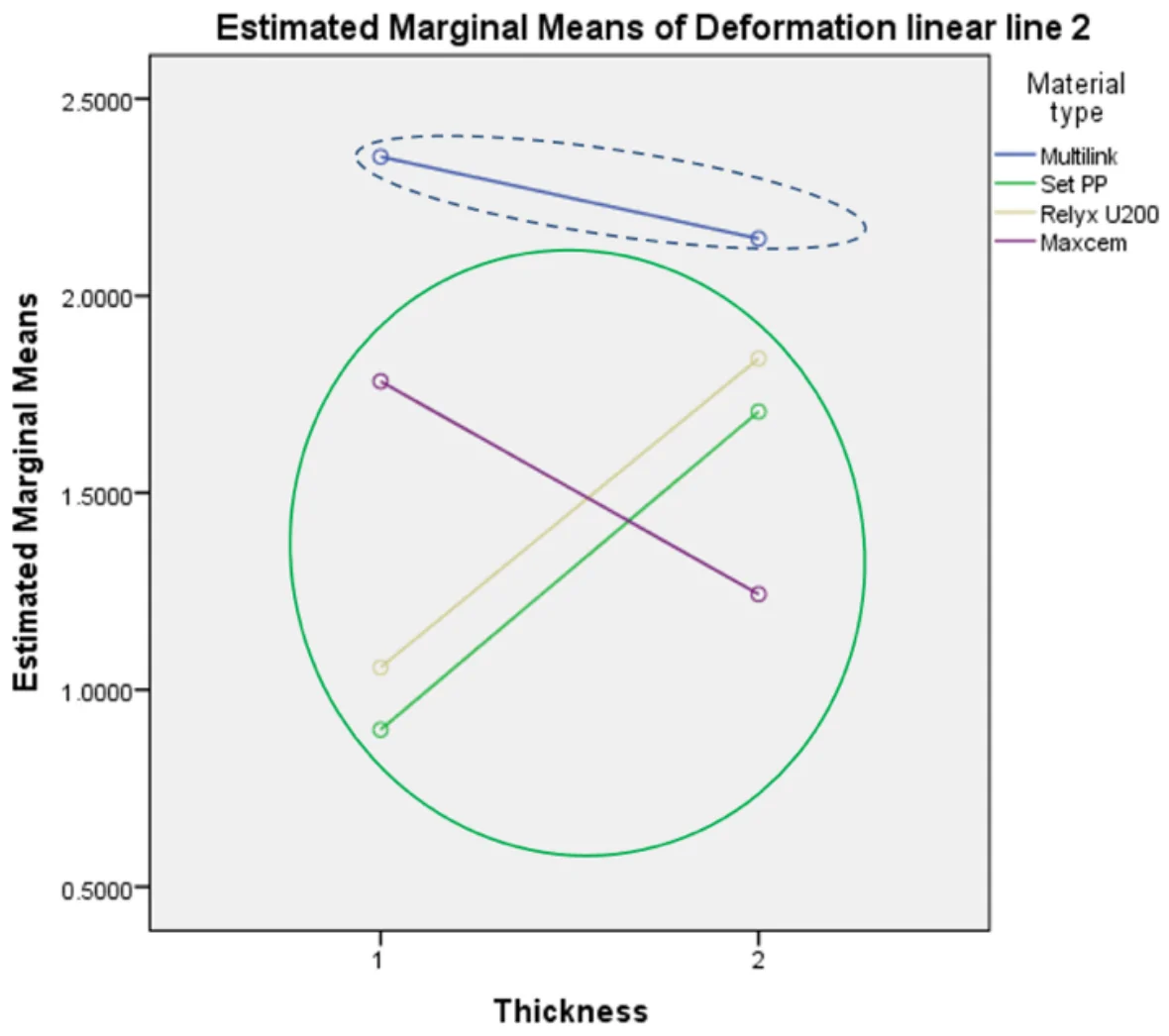
Homogeneous subsets of estimated marginal means of strain values on Section 2.

Using the Tukey’s HSD post hoc test, two groups of homogeneous subsets were obtained depending on the material type and sample thickness, as shown in Table 13 and Figure 5.

The obtained results partially confirmed the first hypothesis. Namely, the strain of four RBCs depends on material type and the combination of material type and sample thickness. The obtained results showed that the combination of material type and sample diameter did not affect the strain, nor did the combination of material type, sample thickness and sample diameter.

When analyzing the strain values, the place where the data are taken from the sample has an influence on the result. The results obtained show that the homogeneous subsets for Section 0 are the Maxcem and SeT PP materials as the first subset, and Multilink and RelyX U200 materials as the second subset. In Sections 1 and 2 in relation to strain, Multilink is observed differently compared with the other three materials (RelyX U200, Maxcem, and SeT PP). The same result was obtained for Sections 1 and 2. This confirmed the last hypothesis, H1.

Based on the obtained results, Multilink has a different behavior in terms of strain values than the other three materials. This difference is reflected in the higher strain values in Multilink. For Multilink, the sample thickness did not significantly affect the strain magnitude.

## 4. Discussion

The combination of three-way ANOVA and Tukey’s HSD post hoc analysis allowed the simultaneous evaluation of material type, specimen thickness, and specimen diameter as determinants of polymerization shrinkage strain in four self-adhesive dual-cure resin cements, measured across three independent sections of the specimen (Sections 0, 1, and 2). Multifactorial analysis is a statistical technique that allows the simultaneous evaluation of several independent variables and their interactions on a single outcome measure. In contrast to univariate designs that examine one factor at a time, a multifactorial approach captures not only the individual (main) effects of each variable but also their combined, interactive effects, offering a more comprehensive and statistically efficient characterization of complex experimental systems. This is particularly relevant in dental materials research, where the mechanical and physical behavior of a material is rarely governed by a single parameter but rather by the interplay of several factors.

Across all three sections, the statistical pattern was consistent: material type exerted a highly significant effect (*p* < 0.001), as did the interaction between material type and specimen thickness (*p* = 0.002 for Section 0, *p* = 0.027 for Section 1, *p* = 0.002 for Section 2). Specimen diameter, whether considered alone or in combination with the other two factors, had no significant effect on strain in any section (*p* > 0.05 throughout), and neither did the three-way interaction of material type, thickness, and diameter. The effect of thickness alone, however, was section-dependent: it reached significance only in Section 0 (*p* < 0.001), while in Sections 1 and 2 its individual contribution was not significant (*p* = 0.323 and *p* = 0.152, respectively), even though its interaction with material type remained significant in both. This indicates that the circular section at the material–mold interface (Section 0) is more sensitive to thickness-related shrinkage effects than the two orthogonal linear sections, which instead reveal the modulating role of thickness only in combination with material composition.

On the basis of these results, the null hypotheses concerning material type (H0_1_), thickness (H0_2_), and their interaction (H0_4_) were rejected, confirming that all three factors significantly influence strain. The hypotheses concerning diameter (H0_3_) and its interactions with material and thickness (H0_5_, H0_6_) were retained, confirming the absence of a diameter effect across the tested range (3–5 mm). The final hypothesis, H1, which proposed that the location at which strain is sampled from the specimen affects the interpretation of material behavior, was also supported: the homogeneous subsets identified by Tukey’s HSD differed between Section 0 and Sections 1/2, indicating that circular and linear sampling geometries are not interchangeable when characterizing shrinkage-induced strain.

The post hoc comparisons reinforce this finding. In Section 0, two homogeneous subsets emerged: Maxcem Elite and SeT PP formed a low-strain group, while Multilink Automix and RelyX U200 formed a high-strain group, with RelyX U200 showing the highest overall mean strain (3.227%) and the combination of RelyX U200 with 2 mm thickness producing the single highest value recorded (4.107%). In Sections 1 and 2, the grouping shifted: Multilink Automix separated from the other three materials, which clustered together as a single homogeneous subset. This shift suggests that the relative ranking of materials by shrinkage strain is not fixed but depends on whether the measurement plane is circular (capturing shrinkage at the material–mold interface) or linear (capturing shrinkage along the specimen body). Taken together, RelyX U200 and Multilink Automix showed the highest strain values in Section 0, Multilink Automix showed the highest values in Sections 1 and 2, and Maxcem Elite and SeT PP belonged to the low-strain subset in all three sections.

This pattern is consistent with the chemical composition of the materials and with established mechanisms of polymerization shrinkage in resin-based cements. As covalent bonds replace intermolecular spacing between monomers during polymerization, the resulting volumetric contraction is intrinsic to the curing reaction itself, and its magnitude is governed primarily by monomer chemistry and filler content [32]. Monomers of higher molecular weight produce comparatively lower shrinkage than low-molecular-weight monomers, and a higher inorganic filler load restricts monomer and radical mobility, which in turn reduces the achievable shrinkage. Filler particles, having a low coefficient of thermal expansion, further dampen volumetric change as their proportion in the polymer matrix increases relative to the resin fraction.

Within this framework, the comparatively low strain values measured for Maxcem Elite are consistent with its formulation. According to manufacturer data, Maxcem Elite contains the acidic monomer GPDM, which contributes to substrate etching and adhesion, and an amine-free initiator system that limits chemical incompatibility between acidic and self-polymerizing components [33]. Its filler system includes fumed silica, a component generally associated with reduced polymerization shrinkage, which is consistent with the low strain observed here. It should be noted, however, that some prior work has reported that Maxcem Elite sustains a low pH for longer than several other self-adhesive cements and may therefore require more time to achieve complete acid–base neutralization [34], which could in principle compromise polymer network formation; this did not translate into elevated strain in the present dataset, suggesting that filler-driven shrinkage control outweighed any disadvantage from delayed acid–base neutralization under the tested conditions.

At the opposite end, the markedly higher strain recorded for Multilink Automix is attributable to its monomer composition rather than to filler content alone. Unlike the three self-adhesive, self-etching cements tested, Multilink Automix is not self-etching, and its formulation reportedly includes hydrophilic low-molecular-weight comonomers such as HEMA (per manufacturer data); monomers of this type are established to increase polymerization shrinkage due to their small molar volume and correspondingly higher density of reacting double bonds per unit volume [32]. The absence of the phosphoric acid-based self-etching components present in MaxCem Elite and SeT PP, combined with differences in filler dispersion, further distinguishes Multilink Automix from the other three materials and helps explain why it forms a separate subset in Sections 1 and 2.

The strain behavior of SeT PP and RelyX U200 may be interpreted in the context of their respective formulations, although the contribution of individual components cannot be established from composition data alone. According to the manufacturer, SeT PP contains UDMA, methacrylate esters with phosphoric acid groups, camphorquinone (CQ), and inorganic fillers, including fluoroaluminosilicate glass and fumed silica. Although UDMA has a higher molecular weight than diluent monomers such as TEGDMA and HEMA, molecular weight alone does not determine the concentration of polymerizable double bonds per unit volume, which also depends on monomer functionality and the overall resin formulation. In the present study, SeT PP consistently belonged to the low-strain subset across all analyzed sections and exhibited the lowest strain at a specimen thickness of 1 mm (1.849%). This result indicates comparatively low measured strain under the experimental conditions employed, but should not be interpreted as direct evidence of lower volumetric polymerization shrinkage. RelyX U200 contains methacrylate monomers with phosphoric acid groups, TEGDMA, and silanated fillers, together with other formulation components. TEGDMA is a relatively low-molecular-weight dimethacrylate commonly used as a reactive diluent in resin formulations. Its presence may be relevant to polymerization behavior; however, the strain response of a commercial cement cannot be attributed to a single monomer independently of the overall formulation and experimental conditions. In the present study, RelyX U200 exhibited the highest mean strain in Section 0 and the most pronounced increase in strain with specimen thickness, from 2.347% at 1 mm to 4.107% at 2 mm. In contrast, its strain values clustered with those of Maxcem Elite and SeT PP in Sections 1 and 2.

The fact that all four cements are dual-cured is itself relevant to the magnitude of strain observed, since the largest fraction of polymerization, and consequently the largest contribution to shrinkage, occurs early in the curing reaction regardless of the subsequent self-cure phase [35]. This has practical implications independent of material selection: light curing should be favored whenever clinically feasible, reserving the self-cure component for situations that genuinely require a slower reaction without compromising the degree of conversion. Prolonged chemical-only polymerization, by contrast, extends the working time during which restorations such as ceramic inlays or prosthetic pieces are vulnerable to displacement, and raises concern over residual monomer release and associated pulpal toxicity [36]. A further limitation affecting the broader interpretation of these results, and of the dental cement literature in general, is that manufacturers frequently disclose only partial formulation data; given the widespread clinical use of these materials, more complete compositional transparency would materially improve the ability of researchers to relate measured mechanical behavior to chemistry with confidence.

From a methodological standpoint, these results carry direct implications for the design of future shrinkage-strain studies on dental resin cements. Sample thickness should be treated as the primary geometrical variable of interest, since its statistically significant main effect in Section 0 and its significant interaction with material type across all sections indicate that 2 mm specimens consistently yield higher strain values and greater discriminating power between materials than 1 mm specimens. Sample diameter, by contrast, can reasonably be selected within the 3–5 mm range based on experimental convenience, as no significant main or interaction effect was detected for this parameter in any section. The consistency of the material type and material–thickness effects across circular and linear measurement geometries, combined with the greater sensitivity of Section 0 to thickness, supports the use of cylindrical specimens of 2 mm thickness and 3–5 mm diameter, with the circular section at the material–mold interface (Section 0) prioritized as the primary evaluation plane, as a standardized protocol for future comparative testing of self-adhesive resin cements.

Clinically, the dependence of strain on specimen thickness, and particularly its pronounced effect at the material–mold interface, suggests that thicker cement layers generate higher shrinkage stresses at the tooth–restoration interface. This has direct bearing on the longevity of indirect restorations, as elevated interfacial shrinkage stress is mechanistically linked to marginal gap formation, micro-leakage, and eventual debonding [37]. Clinicians should therefore aim to minimize cement layer thickness wherever the restoration design and seating allow, since this is the parameter most consistently associated with increased strain across all measurement sections. The absence of a significant diameter effect indicates that restoration size, within clinically relevant dimensions, is a comparatively minor determinant of shrinkage-related risk compared with layer thickness, and clinical attention is therefore better directed at controlling the thickness of the luting layer than at the lateral dimensions of the restoration. Finally, the significant material–thickness interaction indicates that the clinical consequences of cement layer thickness are not uniform across materials: the strain of RelyX U200 increased markedly from 1 to 2 mm, whereas that of Maxcem Elite was practically independent of thickness. Material selection should therefore be considered jointly with the anticipated cement layer thickness. It should also be emphasized that shrinkage strain is only one of the properties that determine clinical performance.

Several limitations of this study should be acknowledged. First, three specimens were tested per factor-level combination. Owing to the multifactorial design, however, each level of the main factors was represented by 18 (material type), 36 (thickness) and 24 (diameter) specimens, and all effects were tested against a common error term with 48 degrees of freedom, which provided adequate sensitivity for effects of the magnitude observed here. The non-significant effect of specimen diameter should nevertheless be interpreted as the absence of a large effect within the tested range rather than as proof of no effect. Second, strain was evaluated immediately after light activation, and the subsequent self-cure contribution of these dual-cured cements was not quantified. Third, the specimens were confined in PTFE molds and were not bonded to tooth tissue or restorative material, so the measured values reflect the shrinkage strain of the cement under standardized constraint rather than the shrinkage stress at a bonded clinical interface.

## 5. Conclusions

Within the limitations of this in vitro study, material type and its interaction with specimen thickness were the dominant factors governing the immediate polymerization shrinkage strain of the four dual-cured resin-based cements, whereas specimen diameter (3–5 mm) had no significant effect. The effect of thickness was material-dependent and most evident at the material–mold interface (Section 0). The location of the evaluation line influenced the ranking of the materials, so circular and linear sections are not interchangeable. For comparative 3D-DIC testing, 2 mm thick specimens evaluated along the circular section at the material–mold interface are recommended, while the specimen diameter can be selected within the 3–5 mm range.

## Figures and Tables

**Table 1:** Experimental design: factors, levels and number of specimens per level (4 × 2 × 3 multifactorial design, *n* = 3 per factor-level combination, 72 specimens in total).

		Value Label	N (Number of Samples)
Material Type	1	Multilink	18
	2	SeT PP	18
	3	RelyX U200	18
	4	Maxcem	18
Thickness	1	1 mm	36
	2	2 mm	36
Diameter	3	3 mm	24
	4	4 mm	24
	5	5 mm	24

**Table 2:** Levene’s test of equality of error variances ^a,b^ for material type and sample thickness.

		Levene Statistics	df1	df2	*p*-Value
Strain of Section 0	Based on mean	1.756	7	64	0.112

Tests the null hypothesis that the error variance of the dependent variable is equal across groups. ^a^ dependent variable: strain on Section 0; ^b^ design: intercept + material type + sample thickness + material type * sample thickness; df1, df2 = the degree of freedom. df1 = number of groups − 1= (8 − 1); df2 = number of cases − number of groups = (72 − 8).

**Table 3:** Tests of between-subjects effects for Section 0.

Source	Type III Sum of Squares	df	Mean Square	F	*p*-Value
Corrected model	44.051 ^a^	23	1.915	4.167	0.000
Intercept	500.552	1	500.552	1089.158	0.000
Material type	16.717	3	5.572	12.125	0.000
Sample thickness	9.547	1	9.547	20.773	0.000
Sample diameter	0.724	2	0.362	0.788	0.460
Material type - sample thickness	7.716	3	2.572	5.597	0.002
Material type - sample diameter	6.095	6	1.016	2.211	0.058
Sample thickness - sample diameter	0.662	2	0.331	0.721	0.492
Material - sample thickness - sample diameter	2.588	6	0.431	0.939	0.476
Error	22.060	48	0.460		
Total	566.663	72			
Corrected total	66.110	71			

^a^ R squared = 0.666 (adjusted R squared = 0.506).

**Table 4:** Strain values on Section 0 related to material type.

Material Type	Mean, %	Std. Error	95% Confidence Interval	
			Lower Bound, %	Upper Bound, %
Multilink	2.996	0.160	2.675	3.317
SeT PP	2.189	0.160	1.868	2.511
RelyX U200	3.227	0.160	2.905	3.548
Maxcem	2.135	0.160	1.814	2.456

**Table 5:** Strain values for all materials in Section 0 related to sample thickness.

Thickness	Mean, %	Std. Error	95% Confidence Interval	
			Lower Bound, %	Upper Bound, %
1	2.273	0.113	2.045	2.500
2	3.001	0.113	2.774	3.228

**Table 6:** Strain values on Section 0 related to sample diameter.

Sample Diameter	Mean, %	Std. Error	95% Confidence Interval	
			Lower Bound, %	Upper Bound, %
3 mm	2.501	0.138	2.223	2.779
4 mm	2.741	0.138	2.463	3.019
5 mm	2.668	0.138	2.390	2.946

**Table 7:** The strain values on Section 0 related to material type and sample thickness.

Material Type	Sample Thickness, mm	Mean, %	Std. Error	95% Confidence Interval	
				Lower Bound, %	Upper Bound, %
Multilink	1	2.735	0.226	2.281	3.189
	2	3.257	0.226	2.802	3.711
SeT PP	1	1.849	0.226	1.394	2.303
	2	2.530	0.226	2.075	2.984
RelyX U200	1	2.347	0.226	1.892	2.801
	2	4.107	0.226	3.652	4.561
Maxcem	1	2.160	0.226	1.706	2.614
	2	2.110	0.226	1.656	2.564

**Table 8:** Multiple comparisons of strain values on Section 0, Tukey’s HSD.

(I) Material Type	(J) Material Type	Mean Difference (I-J), %	Std. Error	*p*-Value	95% Confidence Interval	
					Lower Bound, %	Upper Bound, %
Multilink	SeT PP	0.806472 *	0.2259738	**0.004**	0.205072	1.407873
	RelyX U200	−0.230778	0.2259738	0.738	−0.832178	0.370623
	Maxcem	0.860761 *	0.2259738	**0.002**	0.259361	1.462161
SeT PP	Multilink	−0.806472 *	0.2259738	**0.004**	−1.407873	−0.205072
	RelyX U200	−1.037250 *	0.2259738	**0.000**	−1.638650	−0.435850
	Maxcem	0.054289	0.2259738	0.995	−0.547111	0.655689
RelyX U200	Multilink	0.230778	0.2259738	0.738	−0.370623	0.832178
	SeT PP	1.037250 *	0.2259738	**0.000**	0.435850	1.638650
	Maxcem	1.091539 *	0.2259738	**0.000**	0.490139	1.692939
Maxcem	Multilink	−0.860761 *	0.2259738	**0.002**	−1.462161	−0.259361
	SeT PP	−0.054289	0.2259738	0.995	−0.655689	0.547111
	RelyX U200	−1.091539 *	0.2259738	**0.000**	−1.692939	−0.490139

Based on observed means. The error term is mean square (error) = 0.460. * The mean difference is significant at the 0.05 level.

**Table 9:** Strain values on Section 0, Tukey’s HSD ^a,b^.

Material Type	N	Subset	
		1	2
Maxcem	18	2.135039	
SeT PP	18	2.189328	
Multilink	18		2.995800
RelyX U200	18		3.226578
*p*-value		0.995	0.738

The means of groups in homogeneous subsets are displayed. Based on observed means. The error term is mean square (error) = 0.460. ^a^ Uses the harmonic mean sample size = 18.000. ^b^ Alpha = 0.05.

**Table 10:** Tests of between-subjects effects for Section 1.

Source	Type III Sum of Squares	df	Mean Square	F	*p*-Value
Corrected model	24.129 ^a^	23	1.049	2.286	0.008
Intercept	199.359	1	199.359	434.381	0.000
Material type	10.495	3	3.498	7.623	0.000
Sample thickness	0.457	1	0.457	0.995	0.323
Sample diameter	0.030	2	0.015	0.033	0.968
Material type - sample thickness	4.580	3	1.527	3.326	0.027
Material type - sample diameter	2.728	6	0.455	0.991	0.442
Sample thickness - sample diameter	0.686	2	0.343	0.747	0.479
Material type - sample thickness - sample diameter	5.153	6	0.859	1.871	0.105
Error	22.030	48	0.459		
Total	245.518	72			
Corrected total	46.158	71			

^a^ R squared = 0.523 (adjusted R squared = 0.294).

**Table 11:** Strain values on Section 1, Tukey’s HSD ^a,b^.

Material Type	N	Subset	
		1	2
SeT PP	18	1.270472	
RelyX U200	18	1.512039	
Maxcem	18	1.578728	
Multilink	18		2.294739
*p*-value		0.527	1.000

Means are displayed for groups in homogeneous subsets. Based on observed means. The error term is mean square (error) = 0.459. ^a^ Uses the harmonic mean sample size = 18.000. ^b^ Alpha = 0.05.

**Table 12:** Tests of between-subjects effects for Section 2.

Source	Type III Sum of Squares	df	Mean Square	F	*p*-Value
Corrected model	25.235 ^a^	23	1.097	2.900	0.001
Intercept	190.907	1	190.907	504.656	0.000
Material type	9.666	3	3.222	8.518	0.000
Sample thickness	0.800	1	0.800	2.115	0.152
Sample diameter	0.555	2	0.277	0.733	0.486
Material type - sample thickness	6.406	3	2.135	5.645	0.002
Material type - sample diameter	3.163	6	0.527	1.393	0.237
Sample thickness - sample diameter	1.253	2	0.627	1.657	0.202
Material type - sample thickness - sample diameter	3.391	6	0.565	1.494	0.200
Error	18.158	48	0.378		
Total	234.300	72			
Corrected total	43.393	71			

^a^ R squared = 0.582 (adjusted R squared = 0.381).

**Table 13:** Strain values on Section 2, Tukey’s HSD ^a,b^.

Material Type	N	Subset	
		1	2
SeT PP	18	1.302456	
RelyX U200	18	1.449022	
Maxcem	18	1.512806	
Multilink	18		2.249078
*p*-value		0.735	1.000

The means of groups in homogeneous subsets are displayed. Based on observed means. The error term is mean square (error) = 0.378. ^a^ Uses the harmonic mean sample size = 18.000. ^b^ Alpha = 0.05.

## Data Availability

The original contributions presented in this study are included in the article. Further inquiries can be directed to the corresponding author.

## Abbreviations

The following abbreviations are used in this manuscript:

3D-DIC	3D digital image correlation
RBCs	Resin-based cements
ANOVA	Analysis of variance

## Appendix A. Mean and Standard Deviation of the von Mises Strain Data on Section 0

**Material Type**	**Thickness**	**Diameter**	**Mean, %**	**Std. Deviation**	**N**
Multilink	1	3 mm	2.281067	0.6781104	3
		4 mm	2.554067	0.3745408	3
		5 mm	3.369467	0.4611963	3
		Total	2.734867	0.6661131	9
	2	3 mm	2.993667	0.2701136	3
		4 mm	2.750233	0.4123709	3
		5 mm	4.026300	1.4931134	3
		Total	3.256733	0.9809902	9
	Total	3 mm	2.637367	0.6045309	6
		4 mm	2.652150	0.3683431	6
		5 mm	3.697883	1.0517913	6
		Total	2.995800	0.8565986	18
SeT PP	1	3 mm	2.131400	0.7654646	3
		4 mm	1.618667	0.3665069	3
		5 mm	1.796433	0.0922799	3
		Total	1.848833	0.4827340	9
	2	3 mm	2.281800	1.2817384	3
		4 mm	3.281300	0.7691313	3
		5 mm	2.026367	0.8074630	3
		Total	2.529822	1.0254224	9
	Total	3 mm	2.206600	0.9477879	6
		4 mm	2.449983	1.0581403	6
		5 mm	1.911400	0.5292123	6
		Total	2.189328	0.8527824	18
RelyX U200	1	3 mm	2.328867	0.4769015	3
		4 mm	2.472100	1.2366542	3
		5 mm	2.238733	0.9129488	3
		Total	2.346567	0.8111372	9
	2	3 mm	4.079267	0.4662884	3
		4 mm	4.612100	0.6277202	3
		5 mm	3.628400	0.3406081	3
		Total	4.106589	0.6030962	9
	Total	3 mm	3.204067	1.0474321	6
		4 mm	3.542100	1.4639735	6
		5 mm	2.933567	0.9793609	6
		Total	3.226578	1.1405082	18
Maxcem	1	3 mm	2.173267	0.2528843	3
		4 mm	2.332400	0.1362960	3
		5 mm	1.974133	0.6387040	3
		Total	2.159933	0.3831240	9
	2	3 mm	1.740500	0.3595355	3
		4 mm	2.306367	0.3152715	3
		5 mm	2.283567	0.3043339	3
		Total	2.110144	0.3965804	9
	Total	3 mm	1.956883	0.3653390	6
		4 mm	2.319383	0.2176980	6
		5 mm	2.128850	0.4784866	6
		Total	2.135039	0.3791349	18
Total	1	3 mm	2.228650	0.5000234	12
		4 mm	2.244308	0.6932189	12
		5 mm	2.344692	0.8214813	12
		Total	2.272550	0.6666469	36
	2	3 mm	2.773808	1.1000861	12
		4 mm	3.237500	1.0223737	12
		5 mm	2.991158	1.1641363	12
		Total	3.000822	1.0824430	36
	Total	3 mm	2.501229	0.8808476	24
		4 mm	2.740904	0.9935090	24
		5 mm	2.667925	1.0391888	24
		Total	2.636686	0.9649519	72

## Appendix B. Strain Values on Section 1—Descriptive Statistics

**Material Type**	**Sample Thickness**	**Specimen Diameter**	**Mean, %**	**Std. Deviation**	**N**
Multilink	1	3 mm	1.651700	0.9899206	3
		4 mm	1.950467	0.8885963	3
		5 mm	2.839467	0.2977981	3
		Total	2.147211	0.8665183	9
	2	3 mm	2.982933	1.8836648	3
		4 mm	1.878667	0.3942254	3
		5 mm	2.465200	0.5226102	3
		Total	2.442267	1.1059459	9
	Total	3 mm	2.317317	1.5306564	6
		4 mm	1.914567	0.6160791	6
		5 mm	2.652333	0.4321397	6
		Total	2.294739	0.9756905	18
SeT PP	1	3 mm	0.779467	0.0738722	3
		4 mm	1.010233	0.3971287	3
		5 mm	1.151900	0.6241004	3
		Total	0.980533	0.4057968	9
	2	3 mm	1.839500	0.5557683	3
		4 mm	1.893467	0.5955347	3
		5 mm	.948267	0.2069771	3
		Total	1.560411	0.6228334	9
	Total	3 mm	1.309483	0.6803201	6
		4 mm	1.451850	0.6625552	6
		5 mm	1.050083	0.4305535	6
		Total	1.270472	0.5908071	18
RelyX U200	1	3 mm	1.683033	1.1744076	3
		4 mm	1.366767	1.0619763	3
		5 mm	0.797967	0.2231882	3
		Total	1.282589	0.8888603	9
	2	3 mm	1.481600	0.5275236	3
		4 mm	1.625667	0.3809134	3
		5 mm	2.117200	0.2955260	3
		Total	1.741489	0.4593162	9
	Total	3 mm	1.582317	0.8216924	6
		4 mm	1.496217	0.7275055	6
		5 mm	1.457583	0.7595870	6
		Total	1.512039	0.7258262	18
Maxcem	1	3 mm	1.779433	0.0167046	3
		4 mm	2.165300	0.5626047	3
		5 mm	1.836467	0.5083952	3
		Total	1.927067	0.4199427	9
	2	3 mm	1.287400	0.4056256	3
		4 mm	1.467867	0.0712702	3
		5 mm	.935900	0.3291754	3
		Total	1.230389	0.3526764	9
	Total	3 mm	1.533417	0.3722277	6
		4 mm	1.816583	0.5239899	6
		5 mm	1.386183	0.6245278	6
		Total	1.578728	0.5196141	18
Total	1	3 mm	1.473408	0.7794308	12
		4 mm	1.623192	0.8152329	12
		5 mm	1.656450	0.8966312	12
		Total	1.584350	0.8117862	36
	2	3 mm	1.897858	1.1192506	12
		4 mm	1.716417	0.3935333	12
		5 mm	1.616642	0.7784331	12
		Total	1.743639	0.8042187	36
	Total	3 mm	1.685633	0.9678197	24
		4 mm	1.669804	0.6278448	24
		5 mm	1.636546	0.8214110	24
		Total	1.663994	0.8062998	72

## Appendix C. Strain Values on Section 2—Descriptive Statistics

**Material Type**	**Sample Thickness**	**Specimen Diameter**	**Mean, %**	**Std. Deviation**	**N**
Multilink	1	3 mm	2.112833	0.9083863	3
		4 mm	1.944267	0.5436980	3
		5 mm	3.001667	0.3475574	3
		Total	2.352922	0.7432759	9
	2	3 mm	2.832733	1.3025769	3
		4 mm	1.632900	0.2572949	3
		5 mm	1.970067	0.7203490	3
		Total	2.145233	0.9260869	9
	Total	3 mm	2.472783	1.0789929	6
		4 mm	1.788583	0.4169029	6
		5 mm	2.485867	0.7583793	6
		Total	2.249078	0.8215793	18
SeT PP	1	3 mm	0.914400	0.1064900	3
		4 mm	0.812467	0.1432299	3
		5 mm	0.969600	0.4441588	3
		Total	0.898822	0.2490965	9
	2	3 mm	2.112767	1.1096402	3
		4 mm	2.067167	0.9814825	3
		5 mm	0.938333	0.1031970	3
		Total	1.706089	0.9398242	9
	Total	3 mm	1.513583	0.9632660	6
		4 mm	1.439817	0.9304895	6
		5 mm	0.953967	0.2889013	6
		Total	1.302456	0.7857221	18
RelyX U200	1	3 mm	1.133433	0.4411222	3
		4 mm	1.193067	0.9892870	3
		5 mm	0.844533	0.3176500	3
		Total	1.057011	0.5870320	9
	2	3 mm	1.682033	0.6401648	3
		4 mm	1.691467	0.2917603	3
		5 mm	2.149600	0.3538552	3
		Total	1.841033	0.4567399	9
	Total	3 mm	1.407733	0.5762365	6
		4 mm	1.442267	0.7071392	6
		5 mm	1.497067	0.7755035	6
		Total	1.449022	0.6504231	18
Maxcem	1	3 mm	1.820700	0.3287103	3
		4 mm	1.898033	0.6922660	3
		5 mm	1.630133	0.5977870	3
		Total	1.782956	0.5004161	9
	2	3 mm	1.388633	0.4950964	3
		4 mm	1.482500	0.1738843	3
		5 mm	0.856833	0.0832719	3
		Total	1.242656	0.3949148	9
	Total	3 mm	1.604667	0.4441541	6
		4 mm	1.690267	0.5055567	6
		5 mm	1.243483	0.5701853	6
		Total	1.512806	0.5181784	18
Total	1	3 mm	1.495342	0.6839168	12
		4 mm	1.461958	0.7567191	12
		5 mm	1.611483	0.9701078	12
		Total	1.522928	0.7918165	36
	2	3 mm	2.004042	0.9866740	12
		4 mm	1.718508	0.5086260	12
		5 mm	1.478708	0.7028634	12
		Total	1.733753	0.7680905	36
	Total	3 mm	1.749692	0.8699476	24
		4 mm	1.590233	0.6440184	24
		5 mm	1.545096	0.8312422	24
		Total	1.628340	0.7817714	72

## References

[1] Papazoglou E., Rahiotis C., Kakaboura A., Loukidis M. (2006). Curing efficiency of a photo- and dual-cured resin cement polymerized through 2 ceramics and a resin composite. Int. J. Prosthodont..

[2] Yoshida K., Tsuo Y., Atsuta M. (2006). Bonding of dual-cured resin cement to zirconia ceramic using phosphate acid ester monomer and zirconate coupler. J. Biomed. Mater. Res. B Appl. Biomater..

[3] Ling L., Chen Y., Malyala R. (2024). Assessment of Degree of Conversion and Volumetric Shrinkage of Novel Self-Adhesive Cement. Polymers.

[4] Duarte S.J., Botta A.C., Meire M., Sadan A. (2008). Microtensile bond strengths and scanning electron microscopic evaluation of self-adhesive and self-etch resin cements to intact and etched enamel. J. Prosthet. Dent..

[5] Van Landuyt K.L., Yoshida Y., Hirata I., Snauwaert J., De Munck J., Okazaki M., Suzuki K., Lambrechts P., Van Meerbeek B. (2008). Influence of the chemical structure of functional monomers on their adhesive performance. J. Dent. Res..

[6] Nima G., Makishi P., Fronza B.M., Ferreira P.V.C., Braga R.R., Reis A.F., Giannini M. (2022). Polymerization Kinetics, Shrinkage Stress, and Bond Strength to Dentin of Conventional and Self-adhesive Resin Cements. J. Adhes. Dent..

[7] Giráldez I., Ceballos L., Garrido M.A., Rodríguez J. (2011). Early hardness of self-adhesive resin cements cured under indirect resin composite restorations. J. Esthet. Restor. Dent..

[8] Đorđević M., Karađić B., Jovanić P. (2013). Analysis of adhesion characteristics of liner dental materials. Chem. Ind. Chem. Eng. Q..

[9] Szczesio-Wlodarczyk A., Garoushi S., Vallittu P., Bociong K., Lassila L. (2024). Polymerization shrinkage stress of contemporary dental composites: Comparison of two measurement methods. Dent. Mater. J..

[10] Campaner L.M., Alves Pinto A.B., Demachkia A.M., Paes-Junior T.J.d.A., Pagani C., Borges A.L.S. (2021). Influence of Cement Thickness on the Polymerization Shrinkage Stress of Adhesively Cemented Composite Inlays: Photoelastic and Finite Element Analysis. Oral.

[11] Sokolowski G., Krasowski M., Szczesio-Wlodarczyk A., Konieczny B., Sokolowski J., Bociong K. (2021). The Influence of Cement Layer Thickness on the Stress State of Metal Inlay Restorations Photoelastic Analysis. Materials.

[12] Braga R.R., Ballester R.Y., Ferracane J.L. (2005). Factors involved in the development of polymerization shrinkage stress in resin-composites: A systematic review. Dent. Mater..

[13] Tribst J.P.M., dos Santos A.F.C., da Cruz Santos G., da Silva Leite L.S., Lozada J.C., Silva-Concílio L.R., Baroudi K., Amaral M. (2021). Effect of Cement Layer Thickness on the Immediate and Long-Term Bond Strength and Residual Stress between Lithium Disilicate Glass-Ceramic and Human Dentin. Materials.

[14] Mitrović A., Antonović D., Tanasić I., Mitrović N., Bakić G., Popović D., Milošević M. (2019). 3D Digital Image Correlation Analysis of the Shrinkage Strain in Four Dual Cure Composite Cements. Biomed. Res. Int..

[15] Mitrović A., Tanasić I., Mitrović N., Miletić V., Bakić G., Milošević M., Antonović D. (2019). Analysis of the strain and hardness in self-cured and light-cured self-adhesive resin based cement. J. Adhes. Sci. Technol..

[16] Lezaja M., Veljovic D., Manojlovic D., Milosevic M., Mitrovic N., Janackovic D., Miletic V. (2015). Bond strength of restorative materials to hydroxyapatite inserts and dimensional changes of insert-containing restorations during polymerization. Dent. Mater..

[17] Manojlovic D., Dramićanin M.D., Milosevic M., Zeković I., Cvijović-Alagić I., Mitrovic N., Miletic V. (2016). Effects of a low-shrinkage methacrylate monomer and monoacylphosphine oxide photoinitiator on curing efficiency and mechanical properties of experimental resin-based composites. Mater. Sci. Eng. C.

[18] Bardocz-Veres Z., Miklós M.L., Biró E.K., Kántor É.A., Kántor J., Dudás C., Kerekes-Máthé B. (2024). New Perspectives in Overcoming Bulk-Fill Composite Polymerization Shrinkage: The Impact of Curing Mode and Layering. Dent. J..

[19] Matos J.D.M.d., Queiroz D.A., Nakano L.J.N., Andrade V.C., Ribeiro N.d.C.R., Borges A.L.S., Bottino M.A., Lopes G.d.R.S. (2022). Bioengineering Tools Applied to Dentistry: Validation Methods for In Vitro and In Silico Analysis. Dent. J..

[20] Yoon S., Jung H.J., Knowles J.C., Lee H.H. (2021). Digital image correlation in dental materials and related research: A review. Dent. Mater..

[21] Pratap B., Gupta R.K., Bhardwaj B., Nag M. (2019). Resin based restorative dental materials: Characteristics and future perspectives. Jpn. Dent. Sci. Rev..

[22] Zhang X., Zhang Q., Meng X., Ye Y., Feng D., Xue J., Wang H., Huang H., Wang M., Wang J. (2021). Rheological and Mechanical Properties of Resin-Based Materials Applied in Dental Restorations. Polymers.

[23] Revilla-León M., Meyers M.J., Zandinejad A., Özcan M. (2019). A review on chemical composition, mechanical properties, and manufacturing work flow of additively manufactured current polymers for interim dental restorations. J. Esthet. Restor. Dent..

[24] Ciora E., Miron M., Bojoga D., Lungeanu D., Jivanescu A. (2023). Evaluation of the Pulp Chamber Temperature during Tooth Veneer Preparation Using Burs with Different Degrees of Wear—A Preliminary In Vitro Study. Dent. J..

[25] Mitrovic A., Tanasic I., Mitrovic N., Milosevic M., Tihacek-Sojic L., Antonovic D. (2018). Strain determination of self-adhesive resin cement using 3D digital image correlation method. Serbian Arch. Med..

[26] Dikova T., Maximov J., Todorov V., Georgiev G., Panov V. (2021). Optimization of Photopolymerization Process of Dental Composites. Processes.

[27] Maximov J., Dikova T., Duncheva G., Georgiev G. (2022). Influence of Factors in the Photopolymerization Process on Dental Composites Microhardness. Materials.

[28] Sutton M.A., Orteu J.J., Schreier H.W. (2009). Image Correlation for Shape, Motion and Deformation Measurements: Basic Concepts, Theory and Applications.

[29] Zimmerman D.W. (2004). A note on preliminary tests of equality of variances. Br. J. Math. Stat. Psychol..

[30] Hartung J., Argaç D., Makambi K.H. (2002). Small sample properties of tests on homogeneity in one—Way Anova and Meta—Analysis. Stat. Pap..

[31] Field A.P. (2018). Discovering Statistics Using IBM SPSS Statistics.

[32] Topa-Skwarczyńska M., Ortyl J. (2023). Photopolymerization shrinkage: Strategies for reduction, measurement methods and future insights. Polym. Chem..

[33] Chrisostomo D.A., Strazzi-Sahyon H.B., Briso A.L.F., dos Santos P.H. (2020). Efficacy of Polyacrylic Acid as a Conditioning Agent on the Bond Strength of Self-adhesive Resin Cements to Dental Enamel. Oral Health Prev. Dent..

[34] Zorzin J., Petschelt A., Ebert J., Lohbauer U. (2012). pH neutralization and influence on mechanical strength in self-adhesive resin luting agents. Dent. Mater..

[35] Aldhafyan M., Silikas N., Watts D.C. (2022). Influence of curing modes on conversion and shrinkage of dual-cure resin-cements. Dent. Mater..

[36] Aldhafyan M., Silikas N., Watts D.C. (2022). Influence of curing modes on monomer elution, sorption and solubility of dual-cure resin-cements. Dent. Mater..

[37] Soares C.J., Faria-E-Silva A.L., Rodrigues M.d.P., Fernandes Vilela A.B., Pfeifer C.S., Tantbirojn D., Versluis A. (2017). Polymerization shrinkage stress of composite resins and resin cements—What do we need to know? Braz. Oral Res..

